# Symptom-Checklist-K-9: Norm values and factorial structure in a representative German sample

**DOI:** 10.1371/journal.pone.0213490

**Published:** 2019-04-05

**Authors:** Katja Petrowski, Bjarne Schmalbach, Sören Kliem, Andreas Hinz, Elmar Brähler

**Affiliations:** 1 Medical Psychology and Medical Sociology, University of Mainz, Department Psychosomatic Medicine, Mainz, Germany; 2 University of Münster, Department of Psychology, Münster, Germany; 3 Criminological Research Institute of Lower Saxony, Germany; 4 University of Leipzig, Department of Medical Psychology and Medical Sociology, Leipzig, Germany; 5 University of Mainz, Department Psychosomatic Medicine, Mainz, Germany; Public Library of Science, UNITED KINGDOM

## Abstract

**Background:**

The SCL-K-9 is the latest short version of the multidimensional Symptom-Checklist 90-R. Up to now, its psychometric properties have not been clarified sufficiently as the nine items have not yet been presented exclusively in a representative sample. Therefore, psychometric properties, model fit values as well as norm-values were analyzed.

**Methods:**

For the sample, *N* = 2,507 participants aged 14 to 92, n = 1,379 women and n = 1,128 men, and a mean age of 48.79 (SD = 17.91), were selected from the general population by random-route sampling. Confirmatory factor analyses applying full information maximum likelihood (FIML) tested the model fit. The reliability estimations and effect sizes were reported.

**Results:**

The items’ discriminative power ranged between .49 to .65, and the Cronbach’s Alpha was α = .87, which stands for a good reliability of the SCL-K-9. Norm values as well as gender and age specificities were presented in this section. The CFA with all nine items loading on one latent factor resulted in a good fit. There was evidence of invariance across age and gender groups.

**Summary:**

Based on these results, the short screening version SCL-K-9 of the Symptom-Checklist 90-R showed good reliability and good model fit; specific norm values could be determined. Further studies should evaluate the usefulness of the standardization in clinical samples.

## Introduction

The Symptom-Checklist SCL-90-Revised [1, 2] and its short forms are the multidimensional screening instruments for mental symptoms in psychotherapy research employed most frequently [3, 4, 5]. The instrument includes subscales for depressive, dysthymic, vegetative, agoraphobic, and socio-phobic symptoms as well as symptoms of distrust and a general severity index (GSI). The GSI is considered the best indicator for global psychological distress [6]. Several representative samples with over 4, 500 individuals filled out the Symptom-Checklist SCL-90-Revised [1]. Satisfactory reliability and an almost identical six- factorial structure could be shown consistently [7]. However, statistical test shortcomings in the SCL-90-R were observed in patients with chronic pain [8, 9]. In addition, the allocation of the items to the nine scales was suboptimal in psychiatric patients as well as in healthy individuals [10, 11, 12]. As statistical shortcomings in the SCL-90-R had been observed, several short versions were developed, each with a different number of items [8, 9].

Several short versions of the SCL-90-R were developed, e.g., the Hopkins Symptom Checklist (HSCL-25) [8], the SCL-27 [13], the SCL-5 [9], and the SCL-K-9 [6].

The **HSCL-25** and its norm values were published by Derogatis and colleagues in 1974 [8] and assesses symptoms of anxiety and depression [14]. The reliability was good (Cronbach’s Alpha .84 to .87). The test-retest reliability was satisfactory with a range from .75 to .84. For the evaluation of mental health, the HSCL-25 was compared to a gold standard semi-structured psychiatric interview, the Psychiatric Assessment Schedule (PAS). The HSCL-25 showed only modest properties for the correct assessment of mental disorders [15]. This underlines the need for more efficient questionnaires for measuring mental disorders.

Based on the German version of the SCL-90-R [16], a similar screening version with 27 items (six scales) was developed (**SCL-27**) [17]. In contrast to the HSCL-25, only satisfactory reliability could be shown (Cronbach’s Alpha > 0.70–0.90). However, an almost identical six-factorial structure could be replicated [13]. The factorial structure showed an invariance concerning gender [16], which could not be replicated in later representative samples [13].

Since 25 or 27 items of a short screening of mental health are still too uneconomical for large representative multi-topic evaluations, a further item reduction was undertaken. A **SCL-5** version was developed, which correlates highly with *r* = .92 of the original SCL-25 version [18]. The five anxiety and depression items are treated as a global measure of mental health and are considered emotional distress. The reliability of the SCL-5 was good (Cronbach’s Alpha = .80) [19, 20]. Further psychometric properties as well as norm values of this version were not available. In addition, the selected items and their limited heterogeneity concerning the indicators of global distress were critiqued (by looking at the GSI-90) [21].

In order to have a more efficient screening instrument representing all nine scales of the original SCL-90-R, **the SCL-K-9** was developed [6]. The nine items singled out showed the greatest discriminant power to the average psychological distress level (GSI-90) in a representative survey. From each scale, the item showing the highest correlation coefficient with the GSI-90 (see Table 1) was chosen. The reliability was good (Cronbach’s Alpha = .87). The GSI of the SCL-K-9 (GSI-9-K) correlated highly with the GSI-90 (*r* = .93) [16]. The results of the major components analysis speak for the validity of a single general factor that can resolve 50% of the variance. In reference to the evaluation of the actual clinical status (convergent validity [16]), the **SCL-K-9** shows significant correlations from .36 to .65 with the Hospital Anxiety and Depression Scale (HADS) [22], the Nottingham Health Profile (NHP) [23], and the Whiteley-Index (WI) [24]. This speaks for the primary acquisition of the psychological components by the SCL, whereas the registration of the physical condition is only secondary.

**Table 1:** Overview of the nine items of the SCL-K-9.

Item number	Item
**1**	…Uncontrollable emotional outbursts
**2**	…Finding it difficult to start something
**3**	…Feeling that you worry too much
**4**	…Emotional vulnerability
**5**	…Feeling observed or talked about
**6**	…Feeling uptight or agitated
**7**	…Feeling of heaviness in your arms and legs
**8**	…Feeling nervous when left to yourself
**9**	…Feelings of loneliness even in company

Within the last decade, the SCL-K-9 was employed in numerous studies as a research tool for measuring psychological distress, its sensitivity to changes by gestalt therapy in major depression [25], and by body image intervention in eating disorders [26]. Furthermore, it was applied as an indicator for psychological distress in lung transplant patients [27], fire victims [28], anxiety patients [29] as well as trauma and addiction patients [30].

Since the multidimensionality and the high scale inter-correlations of the SCL-90-R were critiqued, a more practical and applicable, one-dimensional short version with nine items was created for the measurement of the general factor ‘psychological distress’. However, this one-dimensionality has not yet been evaluated nor the invariances tested. Therefore, the aim of the present study is the examination of the dimensionality of the SCL-K-9 on a large German-speaking sample. Also, the multivariate influences on the one-factor structure will be examined for gender and age group. In order to be able to interpret the results of upcoming studies, the norm values of this representative German sample will be displayed.

## Methods

### Sample

**Fig 1:**
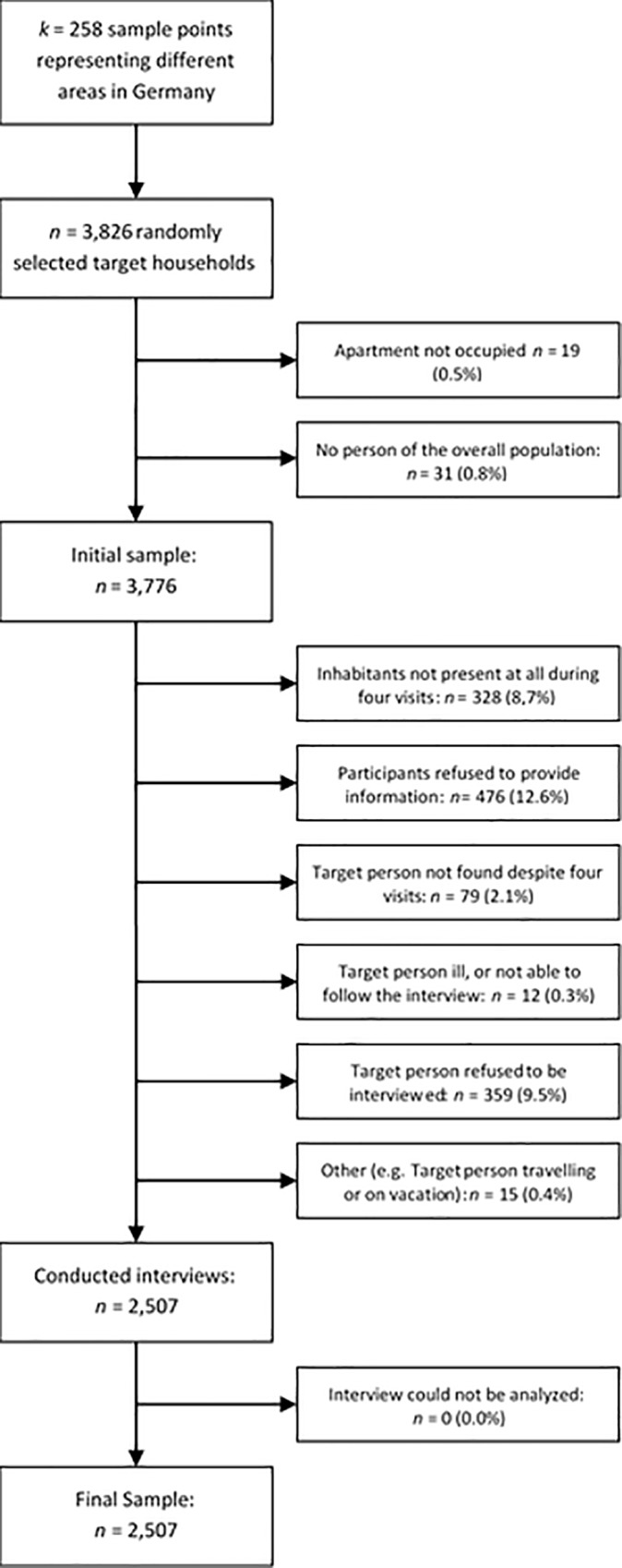
Flowchart of sampling procedure and reasons for nonparticipation.

In 2003, the USUMA (Unabhängiger Service für Umfragen, Methoden und Analysen) Berlin Polling Institute selected households and participants by random-route sampling [31]. The interviews were conducted at the participants´ homes. Reasons for nonparticipation and corresponding figures can be obtained from Fig 1.

Sixty-two percent of all contacted individuals filled out the questionnaire. A final sample of *N* = 2,507 native German speakers who had completed the German SCL-K-9 and the German Patient Health Questionnaire (PHQ-D) were examined (cf. Table 2). Using information from the Federal Statistical Office, the final sample was approved to be truly representative of the German residential population of 2003 in regard to age, gender, and region. All the participants volunteered and received a data protection declaration in agreement with the Helsinki Declaration. Verbal and written informed consent was obtained from all the participants. The study was approved according to the ethical guidelines of the “German Professional Institutions for Social Research” [31] and by the ethic committee of the University of Leipzig (050-13-11032013).

**Table 2:** Sample characteristics concerning sociodemographic variables.

		*N*	%
**Gender**	male	1,128	45.0
	female	1,379	55.0
**Age** (years)	mean	48.79	
(*N* = 2,507; 100%)	standard deviation	17.91	
	range	14 to 92	
**Age groups**	< 24	262	10.5
(years)	25 – 34	332	13.2
(*N* = 2,507; 100%)	35 – 44	485	19.3
	45 – 54	423	16.9
	55 – 64	435	17.4
	65 – 74	386	15.4
	≥ 75	184	7.3
**Marital status**	married, living together	1,304	52.0
(*N* = 2,507; 100%)	married, living separately	42	1.7
	single	615	24.5
	divorced	226	9.0
	widowed	320	12.8
**Education**	not graduated	51	2.0
(*N* = 2,507; 100%)	8^th^ grade	1,126	44.9
	10^th^ grade	816	32.6
	polytechnic degree (without approval as polytechnic degree)	57	2.3
	12^th^/13^th^ grade (Abitur)	191	7.6
	university / college degree	204	8.1
	community college	62	2.5
**Employment status**	full-time (> 35 hours)	922	36.8
(*N* = 2,507; 100%)	part-time (15-35 hours)	193	7.7
	part-time (≤14 hours)	55	2.2
	military/social service; maternity leave	27	1.1
	unemployed	159	6.3
	pensioner	751	30.0
	no longer employed	205	8.2
	in professional training	34	1.4
	in school-/ college education	161	6.4
**Household net income**	< 1,250 € per month	746	29.8
(*N* = 2,368; 94.5%)	1,250 € to 2500 € per month	1,260	50.3
	> 2,500 € per month	362	14.4

### Instruments

The nine- and 27-item versions of the Symptom Checklist (SCL-9-K and SCL-27[6; 13; 17; 21]) measure psychological distress. The SCL-27 assesses global distress and six subscales of specific symptoms: depressive, dysthymic, vegetative, agoraphobic, and socio-phobic, using between four six items on a five-point scale ranging from 0 (*Not at all*) to 4 (*Extremely*) Internal consistency was α ≥ .70 for the subscales and α = .93 for the GSI. The SCL-9-K, on the other hand, is a screener for global symptom severity and does not differentiate between individual types of symptoms. Its internal consistency in a previous study was α = .84 [32].

The Patient Health Questionnaire-D (PHQ-D) was used as an established measure of psychological distress [33]. It allows for the assessment of the severity of the symptoms of depression (α = .88) and somatization (α = .79). Rated on a scale from 0 = (Not at all) to 3 (Almost every day), the participants indicate to what extent a number of symptoms occurred during the two preceding weeks.

### Statistical procedure

The internal consistency of the **SCL-K-9** is reported as Cronbach’s α-coefficient. Item selectivity (discriminatory power) as the correlation of the item with the sum of all other items was determined: item difficulty coefficients were calculated as quotients of the sum of the item values that were obtained and the sum of the maximum achievable item values multiplied by 100.

Shapiro-Wilk was used to test for univariate non-normality on the item level. Gender differences were tested on the item level using Student's paired t-test. In order to quantify the gender differences, we estimated the effect size “g” (ES; Hedges & Olkin, 1985). In accordance with Cohen’s convention (1988), ES > 0.2 is regarded as a small, ES > 0.5 as a medium, and ES > 0.8 as a large effect size.

For the confirmatory factor analysis (CFA), full information maximum likelihood (FIML) [34, 35] estimation was used in order to incorporate the answers from participants with partially missing data. The norm values were based on participants with complete data (*n* = 2,486). CFA was conducted to test the one-factor solution of the SCL-K-9. Given the violation of the multivariate normality assumption, the Yuan and Bentler’s [36] scaled χ^2^ and standard errors (Maximum Likelihood Robust; MLR) [37] were used. MLR, in contrast to the asymptotically distribution-free method (ADF), can also be used on moderately-sized samples without restrictions [38].

To evaluate the goodness of fit of the relevant model, three different criteria were considered: while the root mean square error of approximation (*RMSEA*) as well as the 90% confidence interval assess the absolute model fit, the two additional calculated criteria (Comparative Fit-Index [*CFI*] and the Tucker Lewis Index [*TLI*]) are measurements of a relative model-fit compared to the “null” model. *RMSEA* values < .050 represent a “close fit”, *RMSEA* values between .050 and .080 represent a “reasonably close fit”, and *RMSEA* values > .100 represent an “unacceptable model” [39]. Regarding *CFI* and *TLI*, Hu and Bentler [40] suggested a *CFI* and *TLI* > .950 for a good model fit. The Standardized Root Mean Residual (*SRMR*) generally indicates good fit with values lower than .080 [40].

**Fig 2:**
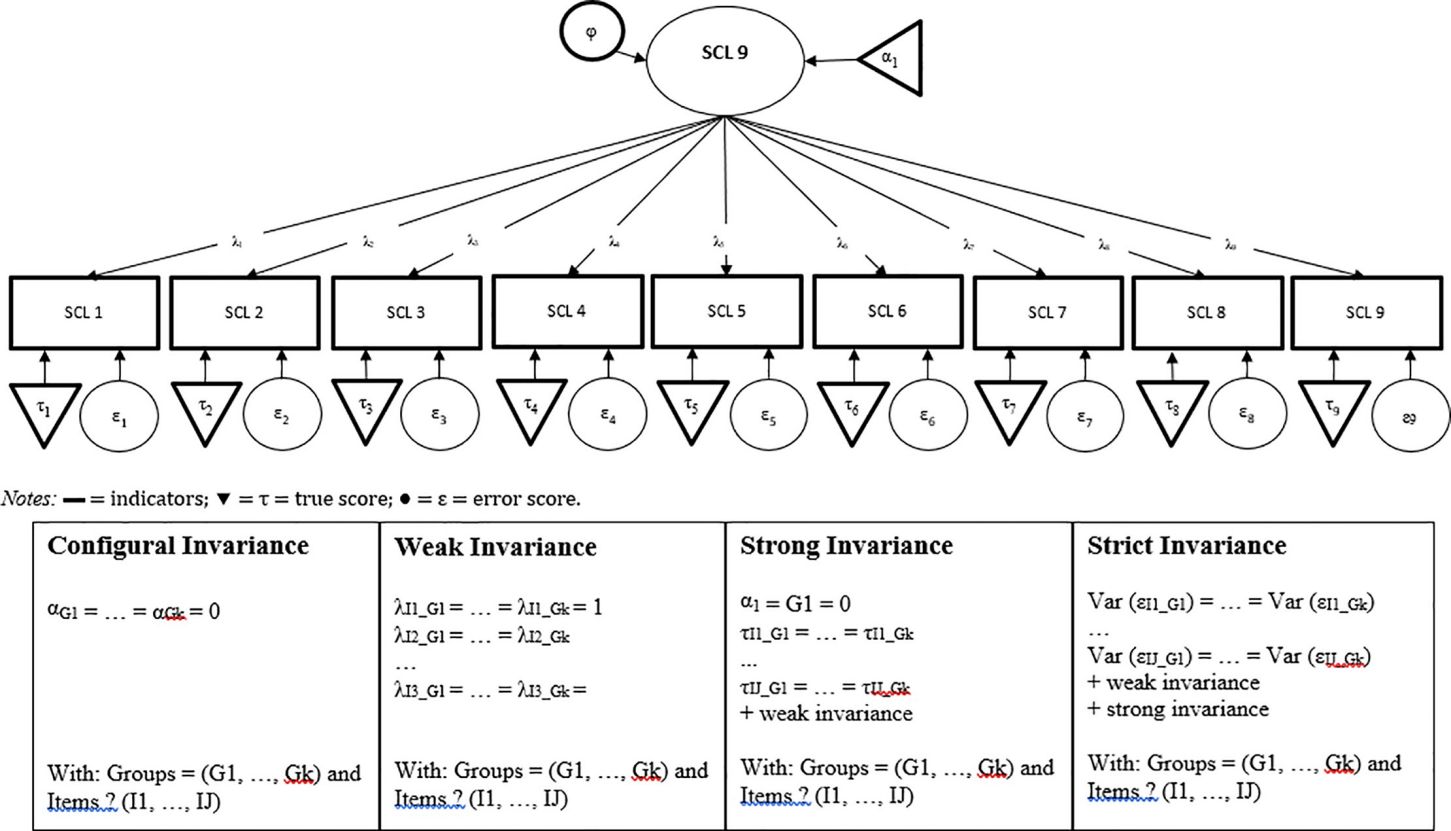
Models relevant for the invariance test.

Furthermore, measurement invariance tests using multi-group factor analyses were conducted across gender (group 1 = men; group 2 = women) and age (group 1: < 25 years of age; group 2: 25 to 34 years of age; group 3: 35 to 44 years of age; group 4: 45 to 54 years of age; group 5: 55 to 64 years of age; group 6: 65 to 74 years of age; group 7: ≥ 75 years of age). Measurement invariance tests were performed using the sequential strategy discussed by Meredith and Teresi [41]: First, a configural invariance model was tested, e.g., which item loads on which factor was imposed on the subgroups. Configural invariance refers to the equivalence of the factorial structure. It is given if the analyzed constructs show the same dimensionality and, in addition, the observed variables are correlated with the same latent constructs in both groups. Configural invariance is necessary but not sufficient for expecting an unbiased comparison of measurements between groups. Second, the weak invariance model was tested by constraining the estimate factor loadings to be equal across groups. If empirical support for weak invariance is provided, it allows the comparison of structural relationships (e.g., correlation coefficients, structural [path] coefficients) between latent constructs in groups. Third, the strong invariance model was tested by constraining both intercepts and loadings to be equal across groups. This level of invariance allows the comparison of means of the latent construct between groups. Finally, the strict invariance model was tested by constraining the loading, intercepts, and item error variances to be equal across groups. Different residual variances in groups may have two possible consequences. First, it may lead to different reliabilities of indices in those groups. Second, it may affect decisions in screening processes that depend on the expression of a construct, resulting in different error rates (e.g. sensitivity, specificity) for different groups [42] (please, see Fig 2 for further details). As noted by Chen [43], the commonly used chi-square differences tests of nested models is almost always significant in large samples and highly sensitive to departures from multivariate normality. Thus, we used scaled *CFI* differences (Δ*CFI*) as well as scaled *RMSEA* differences (Δ*RMSEA*) to compare the difference stages of measurement invariance. As recommended by Chen [43], a change of .010 in Δ*CFI*_scaled_, supplemented by a change of Δ*RMSEA*_scaled_ = 0.015, was regarded as indicative of non-invariance. Furthermore, the absolute model fit of the relevant model was examined using the aforementioned cut-off values. In the case that one or more model parameters identified by invariance tests were found to be variant across samples (partial measurement invariance), the recommendation by Byrne et al. was followed [44] to conduct further invariance tests only when a minimum of two invariant parameters per invariance test (e.g., at least two factor loadings equivalent in metric invariance tests) were found. The data analysis was carried out in R using the packages lavaan and semTools [45, 46].

## Results

### Descriptive item analysis

There were missing data for 21 participants (*n* = 9 male and *n* = 12 female). Therefore, a final sample of N = 2,486 participant was used. As seen in Table 3, item selectivity values range from .49 to .65 and were all above the critical value of 0.3. Significant univariate non-normality was found via the Shapiro-Wilk test with all *W* > .51 (all *p* < .001) as well as for both skewness and kurtosis. Most items tended to be significantly right-skewed and spikier than the Gaussian distribution. The Cronbach’s Alpha was .87, which stands for a good reliability of the SCL-K-9.

**Table 3:** Item characteristics, selectivity and response frequencies of the items.

Item “How much were you bothered or distressed over the past 7 days by …?”	*M (SD)*	Skewness	Kurtosis	selectivity	Alpha without Item	Response Frequencies in %				
						not at all	sometimes	rather intense	intense	very intense
1 … uncontrollable emotional outbursts	0.33 (0.71)	2.96	9.92	.61	.86	76.7%	16.5%	4.4%	1.6%	0.9%
2 … finding it difficult to start something	0.45 (0.78)	2.18	5.26	.63	.85	67.6%	23.3%	6.1%	2.2%	0.8%
3 … feeling that you worry too much	0.64 (0.90)	1.50	2.05	.64	.85	57.5%	27.5%	10.0%	3.7%	1.4%
4 … emotional vulnerability	0.61 (0.89)	1.72	3.00	.65	.85	59.1%	27.4%	8.8%	3.2%	1.1%
5 … feeling observed or talked about	0.38 (0.72)	2.25	5.65	.58	.86	72.8%	19.9%	4.9%	1.8%	0.7%
6 … feeling uptight or agitated	0.53 (0.79)	1.76	3.27	.62	.85	61.3%	27.8%	8.0%	2.3%	0.7%
7 … feeling of heaviness in your arms and legs	0.43 (0.80)	2.03	4.04	.49	.87	71.1%	19.3%	6.1%	2.6%	0.9%
8 … feeling nervous when left to yourself	0.31 (0.68)	2.71	7.75	.61	.86	78.4%	15.0%	4.5%	1.6%	0.5%
9 … feelings of loneliness even in company	0.32 (0.71)	2.62	7.11	.63	.85	78.4%	14.3%	4.7%	2.1%	0.6%
Total	0.40 (0.53)			-	-	-	-	-	-	-

*Notes*: Skewness = third standardized moment representing a measure of distributional asymmetry; Kurtosis = fourth standardized moment, representing a measure of tailedness.

### Effects of gender and age

In total, 1,367 women and 1,119 men responded to all the items of the SCL-K-9. In general, males (*M* = 3.28; *SD* = 4.53) reported lower values in the SCL-K-9 than females did (*M* = 3.91; *SD* = 4.95), *t*(2468.15) = 3.29, *p* = .001, *ES* = 0.13 . Males showed the lowest SCL-K-9 values below 24 years of age. The SCL-K-9 value of males rose continuously with progressing age. Females showed a different pattern concerning the trend of the SCL-K-9 across age groups. Young women (up to 24 years of age) and women older than 65 years of age reported the highest values in the SCL-K-9 questionnaire. In-between these limits females reported lower SCL-K-9 values. The lowest value was found in the age group ranging from 45 to 54.

### Testing of the hypothesized one-factor model and measurement invariance

**Fig 3:**
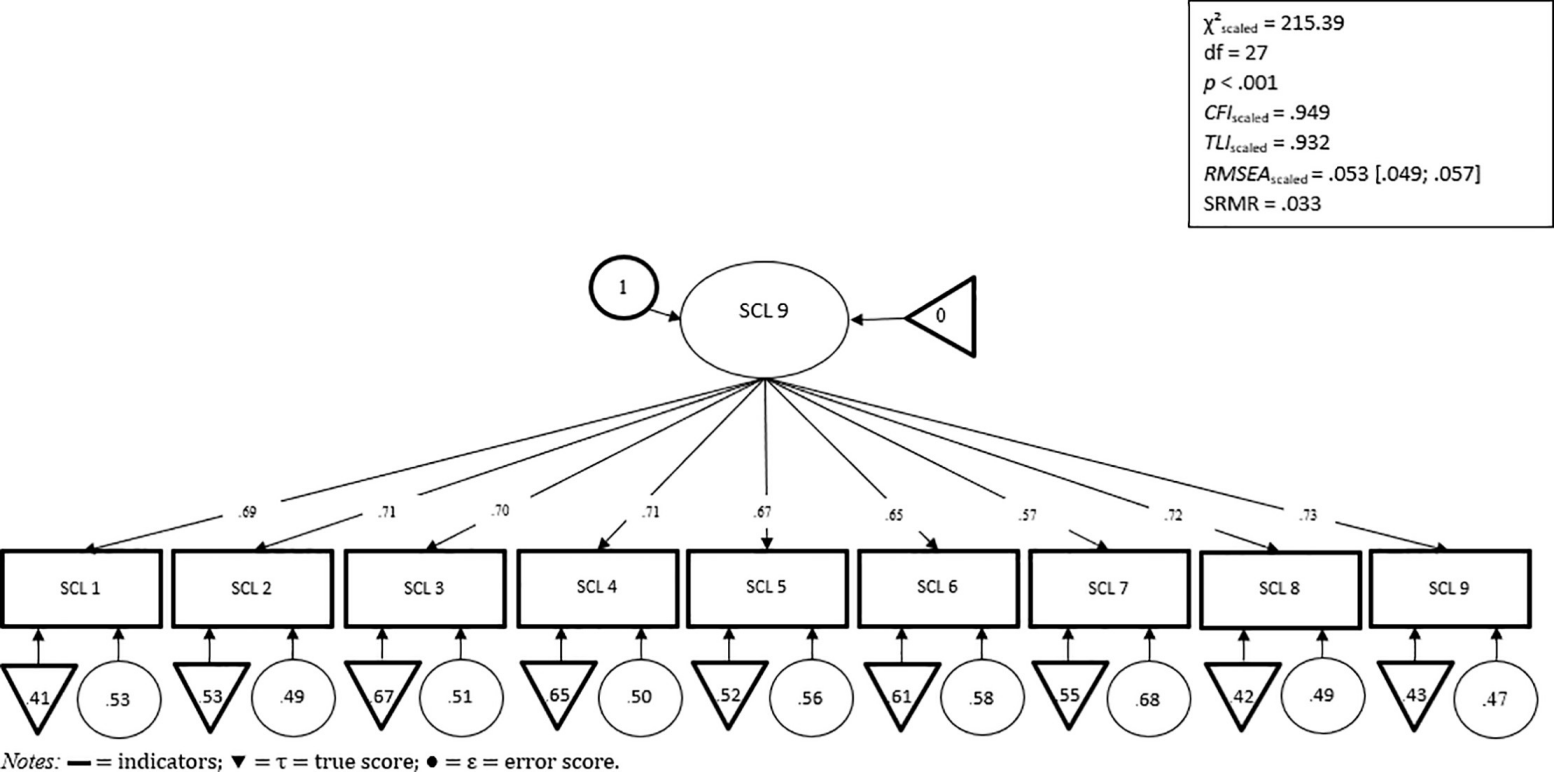
Confirmatory factor analysis model.

Fig 3 shows the results of the CFA for the one-factor solution of the SCL-K-9. An MLR-CFA [36] with all 9 items loading on one latent factor resulted in acceptable to good fit: χ^2^scaled = 215.39, *df* = 27, *p*<.001, *CFI* = .949, *TLI* = .932, *RMSEA*= .053 (90%-CI = .049; .057) and SRMR = .033. Factor loading ranging from λ = .57 to .73.

The results of the measurement invariance analysis regarding age and gender are depicted in Table 4. Regarding gender, the baseline model (Model 0; configural invariance), which simultaneously estimated all model parameters freed across groups, resulted in excellent model fit (*CFI*_scaled_ = .948; *RMSEA*_scaled_ = .054). Weak invariance was examined by comparing Model 0 with Model 1 (see Table 4), which constrained all factor loadings to be invariant across the aforementioned groups. Δ*CFI* and Δ*RMSEA* were below the cut-off recommended by Chen. Furthermore, the model fit was excellent to good (*CFI*_scaled_ = .947; *RMSEA*_scaled_ = .051). Strong invariance was examined by comparing Model 1 with Model 2 (see Table 4), which constrained all the item intercepts to be invariant across groups. Both, Δ*CFI* and Δ*RMSEA* (= .000) were below the cut-off, and the general model fit was excellent to good (*CFI*_scaled_ = .941; *RMSEA*s_caled_ = .051). Therefore, strong invariance can be assumed. Strict invariance was examined by comparing Model 2 with Model 3, which constrained all item residual variances to be invariant across groups. Δ*CFI* and Δ*RMSEA* were below the cut-off recommended by Chen. Furthermore, the model fit was excellent to good (*CFI*_scaled_ = .940; *RMSEA*_scaled_ = .048). Thus, strict invariance can be assumed for gender.

**Table 4:** Analysis of factorial invariance for age and gender using multi-group confirmatory factor analyses.

		*χ^2^*_*scaled*_	*df*	*CFI*	Δ*CFI*	*RMSEA*	Δ*RMSEA*	Measurement Invariance Test^a^
**Gender** (group 1 = men; group 2 = women)								
Model 0	configural invariance	253.16	54	.948	-	.054	-	√
Model 1	weak invariance	262.67	62	.947	.001	.051	.003	√
Model 2	strong invariance	294.49	70	.941	.006	.051	.000	√
Model 3	strict invariance	305.22	79	.940	.001	.048	.003	√
**Age**(group 1: < 25 y; group 2: 25 to 34 y; group 3: 35 to 44 y; group 4: 45 to 54 y; group 5: 55 to 64 y; group 6: 65 to 74 y; group 7: ≥ 75 y)								
Model 0	configural invariance	419.69	189	.945	-	.058	-	√
Model 1	weak invariance	480.26	237	.942	.003	.054	.004	√
Model 2	strong invariance	668.18	285	.909	.033	.061	.007	-
Model 2b	strong invariance (partial)	553.43	273	.934	.008	.054	.000	√
Model 3	strict invariance	705.79	327	.910	.024	.057	.003	-
Model 3b	strict invariance (partial)	625.77	315	.926	.008	.053	.001	√

*Notes*: *df* = degrees of freedom; *CFI* = Comparative Fit Index; Δ*CFI* = differences between models (0 and 1, 1 and 2a; 1 and 2b) regarding *CFI*; *RMSEA* = root mean square of approximation; Δ*RMSEA* = differences between models (0 and 1, 1 and 2a; 1 and 2b) regarding *RMSEA*

^**a**^ **=** *ΔCFI ≥* .*010* supplemented by *ΔRMSEA ≥* .*015* indicates non-invariance. √ marks invariance

Regarding age, the baseline model (Model 0) resulted in an excellent model fit (*CFI*_scaled_ = .945; *RMSEA*_scaled_ = 0.058). Weak invariance was examined by comparing Model 0 with Model 1 (*CFI*_scaled_ = .942; *RMSEA*_scaled_ = .054).; Δ*CFI* and Δ*RMSEA* were below the cut-off recommended by Chen. Strong invariance was examined by comparing Model 1 with Model 2, resulting in a considerable worsening of the model fit (Δ*CFI* = .033) and an unacceptable model fit (*CFI*_scaled_ = .909; *RMSEA*s_caled_ = .061). Subsequently, two-item intercepts were freed between groups (SCL3 “Feeling that you worry too much” and SCL7 “Feeling of heaviness in your arms and legs”). The resulting Model 2b exhibited an acceptable difference in fit compared with Model 1 (Δ*CFI* = .008; Δ*RMSEA* = .000) and a good to excellent model fit (*CFI*_scaled_ = .934; *RMSEA*s_caled_ = .054). Thus, partial strong invariance can be assumed. Strict invariance was examined by comparing Model 2b with Model 3, again resulting in a considerable worsening of the model fit (Δ*CFI* = .024; Δ*RMSEA* = .003). Two-item residual variance was freed between groups (SCL4 ”Emotional vulnerability” and SCL7 ”Feeling of heaviness in your arms and legs”), the resulting Model 3b exhibited an acceptable difference in fit compared to Model 2b (Δ*CFI* = .008; Δ*RMSEA* = .001). Furthermore, the model fit was good to excellent (*CFI* = .926; *RMSEA* = .053). Thus, partial strict invariance can be assumed for the SCL-9 regarding age.

### Validation of the SCL-K-9 version: SCL-27 and PHQ

To analyze whether the SCL-K-9 is an acceptable, efficient tool for identifying mental health, the correlations between the general severity index of the SCL-K-9 and other questionnaires were calculated. The GSI of the SCL-K-9 and the SCL-27 correlated at *r* = .86, which stands for a very high correlation. The correlation coefficient between the GSI-K-9 and scales of the Patient Health Questionnaire were: Stress = .54, Somatic symptoms = .60, and Depression = .71. In this respect, these two questionnaires correlate highly as well.

## Discussion

The SCL-90 [2] is the questionnaire most frequently used internationally to assess psychological distress, especially in clinical practice [47, 48, 49, 50], but it is a very extensive and time-consuming questionnaire. Therefore, short versions were developed for use in large representative studies. One of these is the SCL-K-9 version. The psychometric properties of this version were analyzed in the present study. Internal consistency measured with Cronbach’s Alpha was .87, which stands for a good reliability of the SCL-K-9. Hereby, a low value of alpha could be due to, first of all, a low number of questions, second, a poor interrelatedness between items or, third, a heterogeneous construct. Furthermore, a too high Cronbach’s Alpha value may suggest that some of the items are redundant as the questions refer the same matter but are phrased differently (i.e. item wording). Since in this context a maximum alpha value of 0.90 has been recommended [51], the value determined here may be judged as positive.

Internal consistency is a necessary but insufficient condition for measuring uni-dimensionality in a set of items. Testing the hypothesized one-factor model using MLR-CFA resulted in an acceptable to good model fit. Hence, a unidimensional interpretation of the SCL-9 total score is given and a sum score can be calculated. Furthermore, evidence of strict invariance by sex and age could be found. Therefore, unbiased comparisons of means, correlation coefficients and path coefficients within SEM in multivariable studies are possible, independent of sex and age. Furthermore, undistorted screening of the sex and age groups is possible and explicitly relevant.

Given differences in covariance structure parameters for gender and age, it can be concluded that the SCL-K-9 is a robust instrument for the covariance structure of gender and age in any sample of a multivariable study.

Even though there are fewer items, the SCL-K-9 shows an internal consistency similar to that of other short versions. For example, the HSCL-25 showed a Cronbach’s Alpha range from .84 to .87. However, the ultra-short version SCL-5 showed a lower internal consistency with the Cronbach’s Alpha of .80. Therefore, it can be concluded that the internal consistency of the scale may be affected by a sufficient sample procedure.

The correlation between the GSI-9 and the GSI-90 was calculated as *r* = .93, which stands for a very high correlation. To our knowledge, there has never been another study reporting the associations between the short versions (SCL-K-9) and the full long version (SCL-90-R). With a value of .86, the correlation between the GSI-9 and the GSI-27 as well as the correlation between the GSI-9 and the PHQ-scales Stress = .54, Somatic Symptoms = .60 and Depression = .71, are moderate and high. In a Norwegian sample, the correlations of the SCL-5 with SCL-25 ranged from .91 to .97. In respect to the mental health MHI-5, the correlations were between -.76 and-.78 [9]. These correlations with the SCL-5 were slightly higher than the ones from the present study with the SCL-K-9. This Norwegian sample is also a very large representative sample. However, the sample was drawn over the course of 15 years, therefore, changes over time might have been measured as well. In addition, the SCL-K-9 was implemented by itself, whereby the SCL-5 was taken out of a data set of the longer versions (SCL-25). Hereby, the real associations based on a stand-alone SCL-5 data set and a longer version can only be assumed. Therefore, in comparison to the longer version, these results speak in favor of using the shorter and more efficient SCL-K-9 for assessing mental health.

High interpretation objectivity requires that the findings obtained by an instrument are interpreted in the same way by different diagnosticians. Thereby, it is important that all interpreters possess comparable knowledge regarding the measurements of a questionnaire and how individual or group values are to be interpreted quantitatively. The interpretation of a scale can be subjective if no clear interpretation instructions or reference values/norm values are given in the questionnaire documentation. Without any such information, it can only be said that person or group A has a value B on scale C. In order to interpret, e.g., value Q as high or low, comparison values/standard values are necessary for a representative sample. Therefore, the norm values of the present representative sample were included in the present study (see Tables 5 and 6).

**Table 5:** Normative percentile values: Males.

SCL-9 Sum Score	14–24 (n = 131)	25–34 (n = 142)	35–44 (n = 192)	45–54 (n = 190)	55–64 (n = 208)	65–74 (n = 191)	75–94 (n = 65)
**0**	43.5	40.1	41.1	34.7	33.2	32.5	16.9
**1**	55.0	53.5	52.6	47.9	47.6	46.6	30.8
**2**	62.6	63.4	64.1	58.4	60.6	55.5	44.6
**3**	71.8	77.5	71.4	68.9	67.8	63.4	53.8
**4**	77.9	78.9	77.6	75.3	73.6	70.7	63.1
**5**	80.9	82.4	81.8	80.5	76.9	78.0	69.2
**6**	85.5	85.2	85.9	84.2	82.7	82.2	78.5
**7**	87.0	86.6	87.0	87.9	85.1	85.9	83.1
**8**	90.1	89.4	89.1	88.9	87.5	89.5	84.6
**9**	91.6	91.5	90.6	91.1	89.9	91.1	86.2
**10**	94.7	91.5	91.7	92.6	90.9	91.6	89.2
**11**	94.7	93.0	92.7	93.2	93.3	93.7	89.2
**12**	95.4	94.4	94.3	94.7	95.2	95.8	92.3
**13**	96.2	94.4	94.3	95.8	95.7	96.3	93.8
**14**	96.9	95.8	94.8	96.8	96.6	96.3	93.8
**15**	98.5	96.5	96.4	97.4	96.6	97.9	93.8
**16**	99.2	96.5	96.4	98.4	97.1	98.4	93.8
**17**	99.2	98.6	96.9	98.4	97.1	98.4	93.8
**18**	99.2	99.3	97.4	98.9	97.1	99.0	93.8
**19**	99.2	99.3	97.9	99.5	98.1	99.0	95.4
**20**	99.2	99.3	99.0	99.5	98.1	99.5	95.4
**21**	99.2	99.3	99.5	99.5	99.0	99.5	95.4
**22**	100	100	99.5	99.5	99.5	99.5	95.4
**23**			99.5	99.5	100	99.5	95.4
**24**			100	99.5		100	96.9
**25**				100			100

Note: The standards table is based on a total of 1,119 males.

**Table 6:** Normative percentile values: Females.

SCL-9 Sum Score	14–24 (n = 130)	25–34 (n = 187)	35–44 (n = 291)	45–54 (n = 230)	55–64 (n = 220)	65–74 (n = 191)	75–94 (n = 118)
**0**	30.0	31.6	30.2	32.6	25.5	26.2	20.3
**1**	43.8	43.9	40.9	47.4	40.0	38.7	32.2
**2**	51.5	56.1	53.3	62.2	51.4	48.2	44.1
**3**	63.1	64.7	63.9	68.3	62.3	58.6	54.2
**4**	70.0	72.7	70.1	73.9	67.7	63.9	64.4
**5**	74.6	79.7	75.6	80.0	71.8	71.2	68.6
**6**	79.2	81.8	81.8	85.2	75.0	75.9	72.9
**7**	80.0	85.6	84.9	87.8	80.9	79.1	76.3
**8**	83.1	87.7	87.6	90.0	82.3	82.7	79.7
**9**	85.4	89.3	90.0	92.2	86.8	85.9	82.2
**10**	86.2	90.9	92.4	92.6	90.5	89.0	84.7
**11**	86.9	92.0	93.8	94.3	90.9	90.6	85.6
**12**	87.7	93.0	94.5	95.2	92.7	91.1	89.0
**13**	90.0	94.7	94.8	95.2	95.0	91.6	89.8
**14**	90.8	95.2	95.5	95.2	97.3	91.6	93.2
**15**	92.3	95.2	95.9	97.0	97.7	92.7	94.1
**16**	95.4	95.7	97.3	97.8	98.2	93.2	95.8
**17**	96.9	97.9	97.6	97.8	99.5	94.8	95.8
**18**	97.7	98.4	98.6	98.3	99.5	96.3	96.6
**19**	98.5	99.5	98.6	98.3	99.5	96.3	98.3
**20**	99.2	99.5	98.6	99.1	100	97.4	98.3
**21**	99.2	99.5	98.6	99.6		98.4	99.2
**22**	100	99.5	99.0	99.6		98.4	99.2
**23**		100	99.0	100		98.4	99.2
**24**			99.0			98.4	99.2
**25**			99.0			98.4	99.2
**26**			99.0			99.5	99.2
**27**			99.3			99.5	100
**28**			99.3			100	
**29**			99.3				
**30**			99.3				
**31**			99.3				
**32**			99.3				
**33**			99.3				
**34**			99.3				
**35**			99.3				
**36**			100				

Note: The standards table is based on a total of 1,367 females.

The strength of the present study is its large representative sample and the statistical approach to the results. However, the SCL-K-9 is only a screening instrument, and additional assessments would be necessary for more profound conclusions. The SCL-K-9 enables the screening of mental symptoms in psychotherapy in a time-saving manner. After screening, intervention programs can be implemented more precisely for the population in need, thus avoiding a possible chronification of diseases and their expensive treatment. However, the SCL-K-9 is not suitable for an extensive individual diagnostic as its results merely offer an overview regarding the current psychological state. Therefore, detailed examinations would be called for in the presence of high values.

## Supporting information

S1 Dataset (CSV)

## References

[1] Derogatis LR. SCL-90-R, administration, scoring & procedures manual-I for the R(evised) version. Baltimore: John Hopkins University School of Medicine; 1977.

[2] Derogatis LR. SCL-90-R – Symptom Checkliste 90-R von L.R. Derogatis. Deutsche Fassung von S. Kliem & Elmar Brähler. [Symptom Checklist-90-Revised (SCL-90-R), German version by S. Kliem, & E. Brähler]. Frankfurt am Main: Pearson Assessment & Information; 2017.

[3] Lamborn SD, Monnts NS, Steinberg L, Dornbusch SM. Patterns of competence and adjustment among adolescents from authoritative, authoritarian, indulgent, and neglectful families. Child Dev. 1991;62(5): 1049–1065. 10.2307/1131151 1756655

[4] Perris C, Arrindell WA, Eisemann M. Parenting and psychopathology. New York: Wiley; 1994.

[5] Schumacher J, Eisemann M, Braehler E. Fragebogen zum erinnerten elterlichen Erziehungsverhalten (FEE) [FEE. Questionnaire on the Recalled Parental Rearing Behavior]. Bern: Huber; 2000.

[6] Hessel A, Schumacher J, Geyer M, Brähler E. Symptom-Checkliste SCL-90-R. Diagnostica. 2001;47: 27–39. 10.1026//0012-1924.47.1.27

[7] Fortin MF, Coutu-Wakulczyk G, Engelsmann F. Contribution to the validation of the SCL-90-R in French-speaking women. Health Care Women Int. 1989;10(1): 27–41. 10.1080/07399338909515836 2925532

[8] Derogatis LR, Lipman RS, Rickels K, Uhlenhuth EH, Covi L. The Hopkins Symptom Checklist (HSCL): A self‐report symptom inventory. Behav Sci. 1974;19(1): 1–15. 4808738 10.1002/bs.3830190102

[9] Strand BH, Dalgard OS, Tambs K, Rognerud M. Measuring the mental health status of the Norwegian population: A comparison of the instruments SCL-25, SCL-10, SCL-5 and MHI-5 (SF-36). Nord J Psychiatry. 2003;57(2): 113–118. 10.1080/08039480310000932 12745773

[10] Arrindell WA, van der Ende J. Replicability and invariance of dimensions of parental rearing behaviour: Further Dutch experiences with the EMBU. Pers Individ Dif. 1984;5(6): 671–682. 10.1016/0191-8869(84)90115-6

[11] Parker G, Roussos J, Hadzi-Pavlovic D, Mitchell P, Wilhelm K, Austin, MP. The development of a refined measure of dysfunctional parenting and assessment of its relevance in patients with affective disorders. Psychol Med. 1997;27(5): 1193–1203. 10.1017/S003329179700545X 9300523

[12] Perris C, Jacobsson L, Lindström H, von Knorring L, Perris H. Development of a new inventory for assessing memories of parental rearing behaviour. Acta Psychiatr Scand. 1980;61(4): 265–274. 10.1111/j.1600-0447.1980.tb00581.x. 7446184

[13] Hardt J, Egle UT, Brähler E. Die Symptom-Checkliste-27 in Deutschland: Unterschiede in zwei Repräsentativbefragungen der Jahre 1996 und 2003 [The Symptom Checklist-27 in Germany: Differences between two representative surveys of the years 1996 and 2003]. Psychother Psychosom Med Psychol. 2006;56: 276–84. 10.1055/s-2006-932577 16673337

[14] Glaesmer H, Braehler E, Grande G, Hinz A, Petermann F, Romppel M. The German Version of the Hopkins Symptoms Checklist-25 (HSCL-25) — Factorial structure, psychometric properties, and population-based norms. Comprehensive Psychiatry. 2014;55(2): 396–403. 10.1016/j.comppsych.2013.08.020 24286991

[15] Ventevogel P, Vries GD, Scholte WF, Shinwari NR, Faiz H, Nassery R, et al Properties of the Hopkins Symptom Checklist-25 (HSCL-25) and the Self-Reporting Questionnaire (SRQ-20) as screening instruments used in primary care in Afghanistan. Soc Psychiatry and Psychiat Epidemiol. 2007;42(4): 328–335. 10.1007/s00127-007-0161-8 17370049

[16] Schmitz N, Hartkamp N, Kruse J, Franke GH, Reister G, Tress W. The symptom check-list-90-R (SCL-90-R): a German validation study. Qual Life Res. 2000;9(2): 185–193. 10983482 10.1023/a:1008931926181

[17] Hardt J, Egle UT, Kappis B, Hessel A, Brähler E. Die Symptom-Checkliste SCL-27. PPmP. 2004;54(5): 214–223. 10.1055/s-2003-814786 15106055

[18] Tambs K, Moum T. How well can a few questionnaire items indicate anxiety and depression? Acta psychiatr Scand. 1993;87(5): 364–367. 8517178 10.1111/j.1600-0447.1993.tb03388.x

[19] Brewin C, Andrews B, GoTLIeb IH. Psychopathology and early experience: A reappraisal of retrospective reports. Psychol Bull. 1993;113(1): 82–98. 10.1037//0033-2909.113.1.82 8426875

[20] Matt GE, Vasquez C, Campbell WK. Mood congruent recall of affectively toned stimuli: A meta-analytic review. Clin Psychol Rev. 1992;12(2): 227–255. 10.1016/0272-7358(92)90116-P

[21] Klaghofer R, Brähler E. Konstruktion und Teststatistische Prüfung einer Kurzform der SCL-90–R. Z Klin Psychol Psychiatr Psychother. 2001;49(2): 115–124.

[22] Herrmann-Lingen C, Buss U, Snaith RP. HADS-D - Hospital Anxiety and Depression Scale - Deutsche Version: Ein Fragebogen zur Erfassung von Angst und Depressivität in der somatischen Medizin. Bern: Huber; 1995.

[23] Hinz A, Klaiberg A, Schumacher J, Brähler E. Zur psychometrischen Qualität des Lebensqualitätsfragebogens Nottingham Health Profile (NHP) in der Allgemeinbevölkerung. Psychother Psych Med. 2003;53(8): 353–358. 10.1055/s-2003-40948 12886493

[24] Hinz A, Rief W. Hypochondrie in der Allgemeinbevölkerung: Teststatistische Prüfung und Normierung des Whiteley-Index. Diagnostica. 2003;49: 34–42.

[25] Mittermair F, Singer S. Veränderung von Beschwerdedruck, Kohärenzsinn und Depressivität nach dem gestaltpädagogischen Seminar „Die Heldenreise“. Eine prospektive Interventionsstudie. Musik-, Tanz- und Kunsttherapie. 2008;19(2): 62–69. 10.1026/0933-6885.19.2.62

[26] Vocks S, Legenbauer T, Troje N, Schulte D. Körperbildtherapie bei Essstörungen: Beeinflussung der perzeptiven, kognitiv-affektiven und behavioralen Körperbildkomponente. Z Klin Psychol Psychother. 2006;35: 286–295. 10.1026/1616-3443.35.4.286

[27] Goetzmann L, Irani S, Moser KS, Schwegler K, Stamm M, Spindler A, et al Psychological processing of transplantation in lung recipients: A quantitative study of organ integration and the relationship to the donor. Br J Health Psychol. 2009;14(4): 667–680. 10.1348/135910708X399447 19171083

[28] Ripper S, Renneberg B, Wallis H, Bröking K, Ofer N. Arbeitsfähigkeit, Belastungen und Ressourcen nach schweren Brandverletzungen. Praxis Klinische Verhaltensmedizin und Rehabilitation. 2007;78: 237–243.

[29] Geiser F, Meier C, Wegener I, Imbierowicz K, Conrad R, Liedtke R, et al Association between anxiety and factors of coagulation andfibrinolysis. Psychother Psychosom. 2008;77: 77–383. 10.1159/000151518 18716423

[30] Lüdecke C. Trauma- und Sucht-Therapie In Lüdecke C, Sachsse U, Faure H, editors. Sucht - Bindung - Trauma. Psychotherapie von Sucht und Traumafolgen im neurobiologischen Kontext. Stuttgart: Schattauer; 2010 pp. 175–209.

[31] Arbeitsgemeinschaft ADM-Stichproben Bureau Wendt. Das ADM-Stichprobensystem In Gabler S, Hoffmeyer-Zlotnik JH, Krebs D, editors. Gewichtung in der Umfragepraxis [Samples in survey praxis]. Opladen: Westdeutscher Verlag; 1994 pp. 188–202.

[32] Prinz U, Nutzinger DO, Schulz H, Petermann F, Braukhaus C, Andreas S. Comparative psychometric analyses of the SCL-90-R and its short versions in patients with affective disorders. BMC Psychiatry. 2013;13: 104 10.1186/1471-244X-13-104 23537095 PMC3626675

[33] Gräfe K, Zipfel S, Herzog W, Löwe B. Screening psychischer Störungen mit dem “Gesundheitsfragebogen für Patienten (PHQ-D). Diagnostica. 2004;50(4): 171–181. 10.1026/0012-1924.50.4.171

[34] Enders CK. The impact of nonnormality on full information maximum-likelihood estimation for structural equation models with missing data. Psychol methods. 2001;6: 352–370. 10.1037//I082-989X.6.4.352 11778677

[35] Schafer JL, Graham JW. Missing data: our view of the state of the art. Psychol methods. 2002;7(2): 147–177. 10.1037//1082-989X.7.2.147 12090408

[36] Yuan KH, Bentler PM. Three likelihood‐based methods for mean and covariance structure analysis with nonnormal missing data. Sociol methodol. 2000;30(1): 165–200.

[37] Finney SJ, DiStefano C. Non-normal and categorical data in structural equation modeling In Hancock GR, Mueller RO, editors. Structural Equation Modeling: A Second Course. Greenwich, Conneticut: Information Age Publishing; 2006 pp. 269–314.

[38] Brosseau-Liard PE, Savalei V, Li L. An investigation of the sample performance of two nonnormality corrections for *RMSEA*. Multivariate Behav Res. 2012;47(6): 904–930. 10.1080/00273171.2012.715252 26735008

[39] Browne MW, Cudeck R. Alternative ways of assessing model fit In Bollen KA, Long JS, editors. Testing structural equation models. Newbury Park, CA: Sage; 1993 pp. 136–162.

[40] Hu LT, Bentler PM. Cut-off criteria for fit indexes in covariance structure analysis: Conventional criteria versus new alternatives. Struct Equ Model. 1999;6(1): 1–55. 10.1080/10705519909540118

[41] Meredith W, Teresi JA. An essay on measurement and factorial invariance. Med Care. 2006;44(11): 69–77. 10.1097/01.mlr.0000245438.73837.89 17060838

[42] Millsap RE, Kwok OM. Evaluating the impact of partial factorial invariance on selection in two populations. Psychol Methods. 2004;9(1): 93–115. 10.1037/1082-989X.9.1.93 15053721

[43] Chen FF. Sensitivity of goodness of fit indexes to lack of measurement invariance. Struct Equ Model. 2007;14(3): 464–504.

[44] Byrne BM, Shavelson RJ, Muthén BO. Testing for the Equivalence of Factor Covariance and Mean Structures: The Issue of Partial Measurement Invariance. Psychol Bull. 1989;105(3): 456–466.

[45] Rosseel Y. lavaan: An R Package for Structural Equation Modeling. J Stat Softw. 2012;48(2): 1–36. 10.18637/jss.v048.i02

[46] semTools Contributors. semTools: Useful tools for structural equation modeling. R package version 0.4-14; 2016. Retrieved from https://CRAN.R-project.org/package=semTools

[47] Roth M, Herzberg PY. Psychodiagnostik in der Praxis: State of the Art? Klinische Diagnostik und Evaluation. 2008;1: 5–18.

[48] Christensen J, Fisker A, Lykke Mortensen E, Raabæk Olsen L, Steen Mortensen O, Hartvigsen J, et al Comparison of mental distress in patients with low back pain and a population based referent group. Physiotherapy. 2016;102(1): e182–e183. 10.1016/j.physio.2016.10.218 25964126

[49] Gül AI, Özkırış M, Aydin R, Şimşek G, Saydam L. Coexistence of anxiety sensitivity and psychiatric comorbidities in patients with chronic tinnitus. Neuropsychiatr Dis Treat. 2015;11: 413–418. 10.2147/NDT.S77786 25737637 PMC4344180

[50] Wilgen CP, Vuijk PJ, Kregel J, Voogt L, Meeus M, Descheemaeker F, et al Psychological Distress and Widespread Pain Contribute to the Variance of the Central Sensitization Inventory: A Cross‐Sectional Study in Patients with Chronic Pain. Pain Pract. 2018;18: 239–246. 10.1111/papr.12600 28449376

[51] Streiner D. Starting at the beginning: an introduction to coefficient alpha and internal consistency. Journal of personality assessment. 2003;80:99–103. 10.1207/S15327752JPA8001_18 12584072

